# Dissecting Pseudoaneurysm of the Proper Hepatic Artery Repaired by Primary Anastomosis: A Case Report

**DOI:** 10.1155/2012/804919

**Published:** 2012-11-01

**Authors:** Roshan Razik, Aria Fallah, Charbel Sandroussi, Alice C. Wei, Ian D. McGilvray

**Affiliations:** ^1^Department of Medicine, Toronto General Hospital, 200 Elizabeth Street, Eaton Wing 14-217, Toronto, ON, Canada M5G 2C4; ^2^Division of Neurosurgery, Toronto Western Hospital, 399 Bathurst Street, 4W-436, Toronto, ON, Canada M5T 2S8; ^3^Division of General Surgery, Toronto General Hospital, 200 Elizabeth Street, Eaton Wing 10-215, Toronto, ON, Canada M5G 2C4; ^4^Toronto General Hospital, New Clinical Services Building, 11th Floor Room 11W1250, 585 University Avenue, Toronto, ON, Canada M5G 2N2

## Abstract

*Background*. Although rare, visceral artery pseudoaneurysms often present as surgical emergencies with a specific mortality rate as high as 35% related to aneurysmal rupture. Risk factors for the development of iatrogenic pseudoaneurysms include anticoagulation, female gender, obesity, and vessel calcification. *Case Report*. We present a case of an elderly female who developed a dissecting pseudoaneurysm of the proper hepatic artery after undergoing routine surgery to resect a large duodenal adenoma. Surgical repair comprised of resection and primary anastomosis was employed resulting in a favourable outcome. *Discussion*/*Conclusion*. Surgical management reduces the risk of hepatic ischemia, biliary complications, and abscess formation. Although stenting, coil embolization, and thrombin injection are all plausible options for management, we propose that surgical reconstruction be considered seriously as a treatment for such spontaneous pseudoaneurysms.

## 1. Introduction

The occurrence of pseudoaneurysms in the splanchnic vasculature is very rare with only a few cases being reported in the literature [1, 2]. We report the first case of a pseudoaneurysm of the proper hepatic artery occurring in a postoperative patient and discuss how to choose the appropriate course of management. 

Pseudoaneurysms are defined as a defect through the wall of a blood vessel, including the adventitial layer, causing a localized dilatation [3, 4]. The wall of a pseudoaneurysm is usually comprised of perivascular tissue, thrombus, and reactive fibrosis [5]. Etiologies for pseudoaneurysm formation include trauma, infection, and connective tissue disease [6, 7]. Risk factors for the development of iatrogenic pseudoaneurysms include anticoagulation, female gender, obesity, and vessel calcification. The most common complications from a pseudoaneurysm are due to local compression or rupture [4].

Splanchnic artery aneurysms are uncommon but important, as nearly 22% of these present as surgical emergencies [8]. Approximately one in five splanchnic artery aneurysms involve the hepatic artery with a 2 : 1 predominance in males [6, 9]. The specific mortality rate related to aneurysmal rupture is as high as 35%. 

Biliary tract operative trauma is a rare cause of iatrogenic hepatic artery pseudoaneurysms. Percutaneous intervention is more commonly the inciting event. Clinically, epigastric pain and obstructive jaundice have been reported in symptomatic patients [6].

## 2. Case Report

A 79-year-old female underwent a transduodenal resection of a large adenoma. Preoperative endoscopic biopsy was indeterminate for malignancy, with computed tomography (CT) scan showing a soft tissue mass centered on the second part of the duodenum. There was no evidence of extension to the soft tissue surrounding the duodenum. The hepatic vasculature was normal. She had a past history of intraductal papillary mucinous neoplasm and mild cognitive disease. There was no history of cardiovascular or connective tissue disease. 

Surgery was performed through a midline laparotomy. A longitudinal lateral duodenotomy was performed and the adenoma was isolated. The lesion was excised completely and the duodenum was closed transversely to maintain the lumen. The porta hepatis was not dissected and there was no obvious injury to any of the periduodenal tissue.

**Figure 1:**
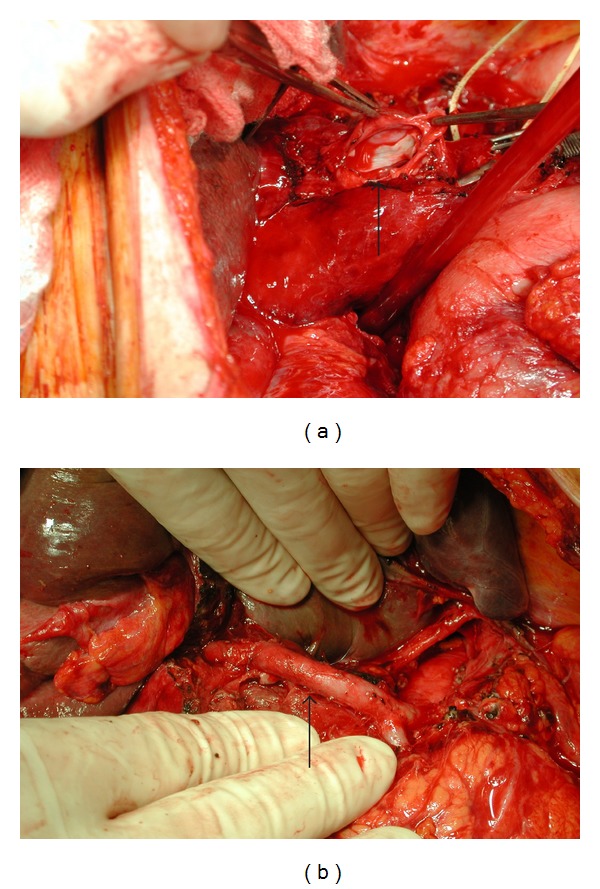
(a) Intraluminal photograph of the PHA pseudoaneurysm (dissected open) after endarterectomy. (b) Photograph of reconstructed PHA. Patency was confirmed by intraoperative Doppler U/S.

**Figure 2:**
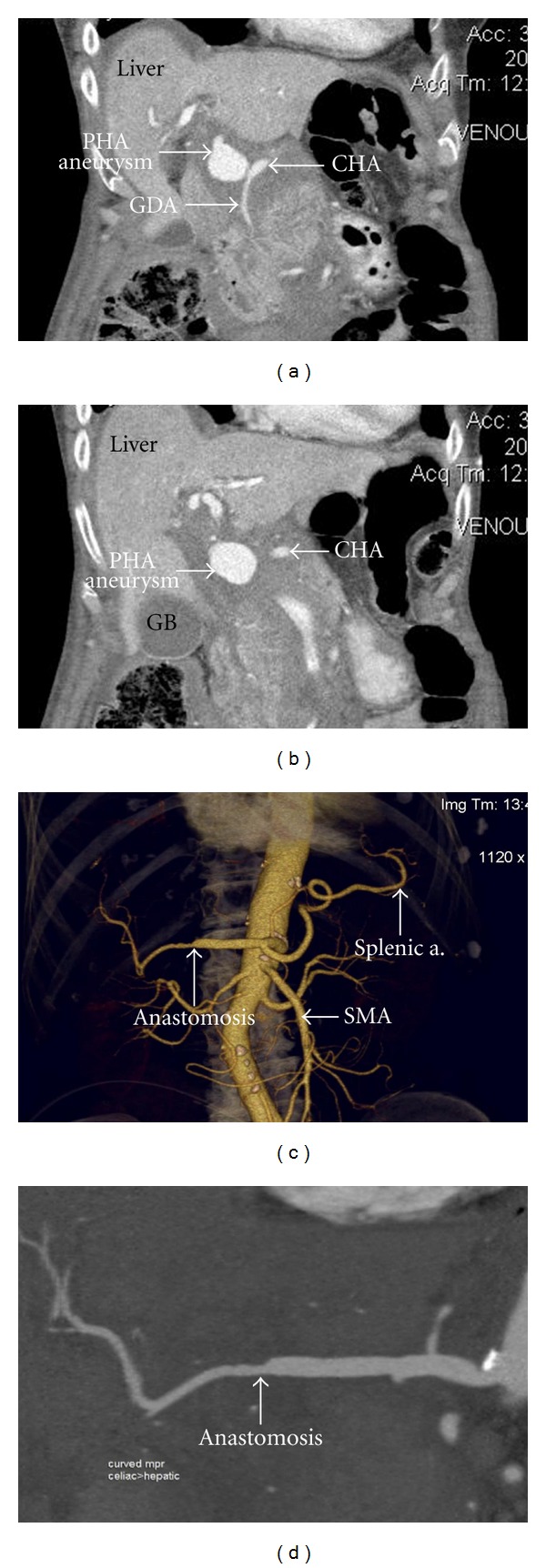
((a), (b)) Coronal CT scan depicting PHA aneurysm extending from the origin of the GDA to the bifurcation of the RHA and LHA. (c) Three dimensional CT reconstruction showing anastomosis between remaining PHA and RHA. (d) Enlarged view of coronal CT scan after anastomosis was completed.

The patient was stable during the immediate postoperative period. On the fourth postoperative day, the patient had increasing serosanguineous output from her Jackson-Pratt drain and a drop in haemoglobin from 128 to 75 g/L. A CT scan was performed which revealed a pseudoaneurysm that appeared to arise from either the gastroduodenal (GDA) or the proper hepatic artery (PHA) measuring 3.1 × 2.5 cm (Figures 1(a), 2(a), and 2(b)). There was minimal free fluid in the peritoneal cavity. It was felt percutaneous treatment with angiographic stenting would present a significant risk for free perforation and would require a long stent with associated increased risk of thrombosis. Thus, angiographic control was not technically possible. 

The patient underwent emergency laparotomy. Proximal and distal control was achieved prior to dissection of the porta hepatis. The inflow to the aneurysm from the common hepatic artery (CHA) was controlled with a bulldog vascular clamp. By following the CHA, the GDA was identified and ligated and the aneurysm localized. A large defect in the adventitia was identified on the right lateral aspect of the PHA.

Rather than simply ligating the PHA, the aneurysm was resected from the origin of the GDA until the bifurcation of the RHA and LHA. On inspection of these vessels, the extrahepatic RHA appeared normal and the LHA had an intimal dissection extending into the liver. The LHA was ligated and reconstruction to the origin of the RHA was performed using microvascular techniques. 

On release of the arterial clamps there was a good pulse and thrill in the RHA. Intraoperative Doppler ultrasound showed a normal triphasic signal in the anastomosis (Figure 1(b)). Post-operative Doppler and CT scan confirmed patency of the reconstruction (Figures 2(c), 2(d)) and adequate perfusion of the liver with only a portion of segment IV showing signs of ischemia. 

The patient was stable postoperatively, had no further complications and was discharged home on postoperative day 11.

## 3. Discussion

Pseudoaneurysm formation in the hepatic arteries is an extremely rare complication of manipulation of the hepatic vasculature during abdominal surgery [10]. In our case, it likely occurred secondary to an occult injury to the artery during normal retraction of the porta hepatis tissues. Barring publication bias, this is the first reported case of pseudoaneurysm occurring postduodenotomy [11, 12]. Although rare, the case raises the issue of the appropriate management of hepatic arterial injury in general: ligation or repair? 

Hepatic artery aneurysms can present with dyspepsia, jaundice, or bleeding [13]. In the perioperative period these signs and symptoms are unreliable and hence a high index of suspicion needs to be maintained in order to make the diagnosis. CT is the modality of choice for diagnosis and in equivocal cases angiography is diagnostic.

Management of aneurysmal disease in the splanchnic vasculature is a relatively new phenomenon. Small asymptomatic pseudoaneurysms (less than 1.0 cm in diameter) can usually be observed. When treatment is warranted, there are several options. First, ultrasound-guided thrombin injection results in instant thrombosis of the pseudoaneurysm under direct visualization. It should be noted that such injections have been performed successfully in the femoral location but no data exists for the splanchnic circulation. Risks include arterial embolization or thrombosis of the parent blood vessel and rarely, anaphylaxis. This procedure is not recommended in unstable aneurysms [14–16]. 

A second treatment modality is coil embolization which utilizes angiography to deploy flexible wires to directly obliterate the pseudoaneurysm. Its benefits include its use in regions where direct puncture percutaneous access is difficult and also in patients that cannot tolerate open surgery. Its disadvantages include contrast-induced nephropathy and significant radiation exposure [2, 17]. This procedure is generally attempted in treating pseudoaneurysms occurring in an artery without collaterals since retrograde flow from collaterals could potentially nullify this intervention [18].

A third treatment option is stenting, whereby a stent graft is introduced using endovascular techniques. The stent effectively excludes the aneurysm from the circulation while maintaining distal flow. This method is preferred for wide-neck aneurysms, large diameter arteries, or vessels with minimal tortuosity [18]. Complications include endoleaks, infection, and treatment failure requiring open surgical repair [12, 19].

Finally, surgical repair of these pseudoaneurysms should be considered seriously depending on the context. Several modalities exist including ligation without arterial reconstruction, patch repair, and primary anastomosis [7, 19]. Simple ligation is commonly the preferred option as collateral circulation to the liver is usually adequate. However, we propose that vascular reconstruction become the norm as this may improve postoperative outcomes, reducing hepatic ischemia and the potential for subsequent biliary complications [19]. In this patient, reconstruction successfully removed the risk of rebleeding and provided adequate arterial inflow to the liver, including reperfusion of the LHA by collaterals. Additionally, had flow not been reestablished to the RHA by primary anastomosis, the risk of hepatic abscess formation would have been seven times higher (14% compared to 2% in non-RHA-compromised patients) [20]. Surgery allows direct visualization but can be limited due to its invasive nature and potential for further vascular injury due to handling of vasculature. 

Although a rare occurrence, it is important for both general and vascular surgeons to consider visceral artery pseudoaneurysm formation as a rare cause of postoperative hemorrhage in patients undergoing upper gastrointestinal surgery. This case demonstrates the feasibility of surgical reconstruction as a primary modality of treatment. We advocate that resection and primary anastomosis be considered as an important treatment option as it ensures optimal perfusion to the liver and likely improved patient outcomes.
